# Finding the fitting framework for the implementation of policies promoting healthy nutrition and physically active lifestyle: Results from a systematic review

**DOI:** 10.1093/eurpub/ckac129.268

**Published:** 2022-10-25

**Authors:** S Forberger, K Lobczowska, A Banik, P Romaniuk, T Kubiak, B Meshkovska, A Neumann-Podczaska, M Kaczmarek, M Scheidmeir, al

**Affiliations:** 1 Leibniz Institute for Prevention Research and Epidemiology, BIPS, Bremen, Germany; 2 Psychology Department, SWPS University of Social Sciences and Humanities, Wroclaw, Poland; 3 Department of Health Policy, Medical University of Silesia in Katowice, Bytom, Poland; 4 Institute of Psychology, Johannes Gutenberg University Mainz, Mainz, Germany; 5 Department of Nutrition, University of Oslo, Oslo, Norway; 6 Department of Palliative Medicine, Poznan University of Medical Sciences, Poznan, Poland

## Abstract

**Background:**

Policies are an important upstream component of health promotion to influence the general population. Various frameworks exist to help implement policies. However, there is currently no overarching synthesis that describes the differences between policy implementation frameworks. In this study, we examined frameworks for implementing policies to promote healthy eating, physical activity and reduce physical inactivity and aimed to explore the scope of the frameworks, the content of the constructs they contain (e.g. processes, determinants, evaluation), the level at which these constructs operate and the inclusion of equity factors.

**Methods:**

A systematic review (PROSPERO registration number: CRD42019133251) was conducted using 9 databases and 8 stakeholder websites. The content of 38 policy implementation frameworks was coded and analysed.

**Results:**

All three constructs were covered by 18 frameworks: description of the process, determinants and evaluation of implementation. The majority of frameworks (25/38) considered constructs from three levels: the individual, organisational/community and system levels, with system level constructs being included less frequently than individual level or organisational/community level constructs. Most frameworks (32/38) contained sections that were exclusively descriptive. In addition, 19 frameworks contained prescriptive and 23 explanatory sections. The complex systems approach was included in 8 of the frameworks. More than half of the frameworks (21/38) did not consider equity constructs.

**Conclusions:**

Most frameworks have a complex scope, include multi-level constructs, combine sections that are purely descriptive with sections that consider prescriptive and/or explanatory associations, and include few or no equity constructs. The findings of this study can facilitate the process of selecting the framework that best fits the needs and goals of policy makers, researchers and policy implementation actors seeking guidance.

